# Effects of the Tangningtongluo formula as an alternative strategy for diabetics via upregulation of insulin receptor substrate-1

**DOI:** 10.3892/mmr.2019.10649

**Published:** 2019-09-04

**Authors:** Long Cheng, Junmei Song, Geng Li, Yue Liu, Yuming Wang, Xiangbao Meng, Guibo Sun, Xiaobo Sun

Mol Med Rep 16: 703-709, 2017; DOI: 10.3892/mmr.2017.6679

Subsequently to the publication of this article, the authors have realized that Fig. 4 contained some incorrectly incorporated data panels; specifically, the A-d, B-b and B-c panels did not display the correct data. The revised version of Fig. 4 featuring the correct data for the A-d, B-b and B-c panels is shown on the next page.

Note that these errors did not affect the overall conclusions reported in the paper. The authors apologize to the Editor of *Molecular Medicine Reports* and to readership for any inconvenience caused.

## Figures and Tables

**Figure 4.**
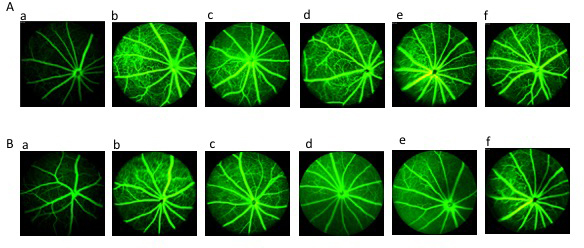
Retinal imaging using FA in each group. (A) Results of the fundus examination using FA prior to drug intervention in the (a) normal, (b) DM, (c) Met, (d) TNTL-h, (e) TNTL-m and (f) TNTL-1 groups. (B) Results of the fundus examination using FA after the 18-week drug intervention in the (a) normal, (b) DM, (c) Met, (d) TNTL-h, (e) TNTL-m and (f) TNTL-1 groups. Magnification, ×40. Normal, C57BL mice; DM, untreated C57BLdb/db mice; Met, C57BLdb/db mice treated with metformin; TNTL-h, TNTL-m and TNTL-l, C57BLdb/db mice treated with 1.8, 0.9 and 0.45 g/kg body weight Tangningtongluo, respectively. FA, fluorescein angiography.

